# From Raman to SESORRS: moving deeper into cancer detection and treatment monitoring

**DOI:** 10.1039/d1cc04805h

**Published:** 2021-11-04

**Authors:** Sian Sloan-Dennison, Stacey Laing, Duncan Graham, Karen Faulds

**Affiliations:** Department of Pure and Applied Chemistry, Technology and Innovation Centre, University of Strathclyde 99 George Street Glasgow G1 1RD UK karen.faulds@strath.ac.uk

## Abstract

Raman spectroscopy is a non-invasive technique that allows specific chemical information to be obtained from various types of sample. The detailed molecular information that is present in Raman spectra permits monitoring of biochemical changes that occur in diseases, such as cancer, and can be used for the early detection and diagnosis of the disease, for monitoring treatment, and to distinguish between cancerous and non-cancerous biological samples. Several techniques have been developed to enhance the capabilities of Raman spectroscopy by improving detection sensitivity, reducing imaging times and increasing the potential applicability for *in vivo* analysis. The different Raman techniques each have their own advantages that can accommodate the alternative detection formats, allowing the techniques to be applied in several ways for the detection and diagnosis of cancer. This feature article discusses the various forms of Raman spectroscopy, how they have been applied for cancer detection, and the adaptation of the techniques towards their use for *in vivo* cancer detection and in clinical diagnostics. Despite the advances in Raman spectroscopy, the clinical application of the technique is still limited and certain challenges must be overcome to enable clinical translation. We provide an outlook on the future of the techniques in this area and what we believe is required to allow the potential of Raman spectroscopy to be achieved for clinical cancer diagnostics.

## Abstract 2

This article discusses the use of various Raman spectroscopy techniques and how they have been applied to different methods of detection for the diagnosis of cancer and in monitoring cancer treatment, with an outlook on how they can progress into a clinical environment.

## Graphical abstract

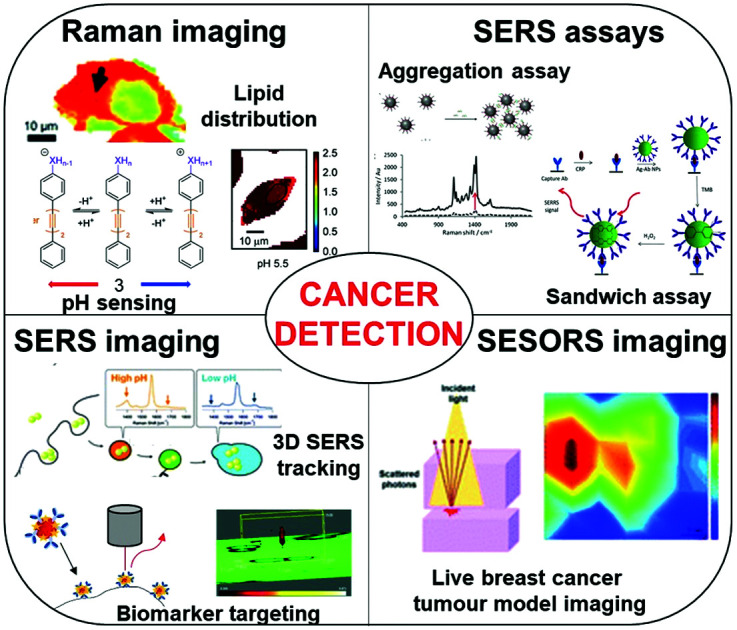

## 1. Introduction

Cancer is the second most common cause of death globally, accounting for an estimated 9.6 million deaths in 2018.^1^ It results from the abnormal proliferation of normal cells in a multi-stage process, resulting in malignant tumours that can invade other parts of the body.^2^ Physical, chemical and biological carcinogens are responsible for the onset of the disease, with prevalence increasing with age as risk factors grow and cellular repair mechanisms become less effective. Therapeutics include invasive surgery, chemotherapy and radiotherapy, which can be ineffective in the later stages. Too often, similar symptoms are observed in benign and malignant cases and end with a low positive predictive value (PPV). However, a symptom combined with a positive test result or a relative clinical finding increases the PPV and shortens the time interval between consultation and treatment.^3^ Despite significant advances in recent years, the early diagnosis and treatment of cancer remains a challenge in medicine. Due to the worldwide prevalence of the disease, and the resulting mortality rates, early detection of cancer is of utmost importance to improve prognosis and patient survival. Non-invasive strategies for early detection, diagnosis and treatment monitoring are therefore urgently needed, and significant progress has been made using Raman spectroscopy and associated enhancement techniques to address these needs.

Raman scattering is a non-invasive technique that has the ability to specifically determine the chemical composition of samples based on the inelastic scattering of light by molecules.^4^ This molecular “fingerprinting” can be used for the sensitive and specific detection of biochemical changes that occur in diseases and is therefore a useful tool for cancer detection, diagnosis and treatment monitoring.^5,6^ Raman spectroscopy in cancer diagnostics has investigated a multitude of different cancer types including lung,^7^ cervix,^8^ breast,^9^ prostate,^10^ lymph nodes,^11^ esophagus,^12^ colon,^13^ larynx,^14^ bladder^15^ and brain.^16^ It has also been used to distinguish between cancerous and non-cancerous samples in *ex vivo* biopsies,^17–19^*in vitro* biomarker detection,^20–22^ and *in vivo* analysis.^16,23–26^ Advancements in instrumentation, such as the development of Raman microscopy, have allowed the technique to be used to produce high resolution chemical images of a sample by collecting spectra across several points of a defined area. These can then be constructed into false colour images using relative intensities of Raman peaks, or specific spectral regions of certain components, allowing the visualisation of changes in the sample based on their chemical properties.^27^ This has been exploited extensively for the label-free detection of biochemical changes in cell and tissue samples.^28–31^ One of the major drawbacks of Raman scattering is that signals are inherently weak due to the small proportion of photons that are inelastically scattered. This often results in poor signal to noise, reducing the sensitivity of the technique. The spectra are also complicated and often require further multivariate analysis techniques to deconvolute the data.

One method of improving the sensitivity of Raman scattering is the use of non-linear Raman techniques, such as coherent anti-Stokes Raman scattering (CARS)^32,33^ or stimulated Raman scattering (SRS).^34^ CARS and SRS are multiphoton systems where two excitation lasers, the “pump” and the “Stokes”, are used to excite specific vibrational modes within a sample. In CARS imaging, one laser frequency is fixed (*ν*_S_) and the other (*ν*_P_) is tuned to excite a specific molecular vibration. The interaction of the two laser beams results in anti-Stokes photons of frequency *ν*_AS_ = 2*ν*_P_ − *ν*_S_ and the corresponding anti-Stokes signal is detected to produce a CARS image. However, CARS suffers from a non-resonant background that interferes with the resonant vibrational signal and reduces image contrast, leading to distorted line shapes that decrease the amount of chemical information, resulting in data that is difficult to interpret.^35^

In SRS, where the difference in frequency between the “pump” and “Stokes” photons matches the frequency of a molecular vibration (*ν*_vib_ = *ν*_P_ − *ν*_S_), excitation of the vibration is stimulated and small beam intensity changes can be detected to produce images at the selected frequency (*ν*_vib_). The SRS signal of a molecular species is linearly proportional to its concentration, whereas in CARS it is proportional to the square of the concentration and the laser power. Therefore, SRS has greater potential to be a powerful method for label-free quantitative determination of individual species in a multi-component system. SRS images are also free from non-resonant background and the spectra obtained match the Raman spectra of the sample, making the chemical data easily interpretable.^36^

Non-linear Raman techniques offer greater spatial resolution and rapid imaging times in comparison to conventional Raman spectroscopy. Generally, CARS and SRS are used to study cellular components such as lipids, DNA and proteins, and the techniques can be used to obtain detailed images, where cell structure and morphology can be examined. CARS and SRS have been employed to investigate cellular changes for the detection and diagnosis of cancer,^37–41^ and to monitor the uptake of drugs by cancer cells.^42^ SRS is also used to image small molecules coupled to vibrational tags such as nitriles or alkynes.^43^ Alkynes are preferred due to the C≡C stretching motion that exhibits a substantial change in polarisability, producing a sharp Raman peak in the cell silent region.^44^ Proteins, DNA and phospholipids have all been tagged with alkynes, introduced into cells and imaged using SRS, offering superb sensitivity, specificity and the biocompatibility required to study complex living systems.^45,46^

Further enhancements of weak Raman signals can be achieved by introducing a roughened metal surface. This phenomenon, known as surface enhanced Raman scattering (SERS), occurs when a molecule is adsorbed onto, or held in close proximity to, an enhancing metal surface.^47,48^ SERS enhancement is a result of the interaction of light with plasmons excited at the surface of the metal, which has been shown to enhance Raman signals up to 10^10^.^49^ Nanoparticles of noble metals (most commonly gold and silver) are used as SERS substrates due to their unique optical properties and adaptable synthesis allowing control over size, shape, and morphology, which can be tailored towards diagnostic applications. SERS-active nanoparticles can either be used in a label free (direct) capacity, where the intrinsic scattering from a biomolecule of interest adsorbed onto a nanoparticle surface is obtained, or for labelled (indirect) detection, which is achieved when Raman reporters are added to the nanoparticle surface to create SERS nanotags that can be used to indirectly detect biomolecules.^50^ Further signal enhancement can also be achieved when the Raman reporter is a chromophore with an electronic transition close in energy to the exciting laser. This increased enhancement is known as surface enhanced resonance Raman scattering (SERRS), which has been reported to increase signals up to 10^14^.^51^ SE(R)RS nanotags can also have targeting capabilities by functionalising them with biomolecules, offering further potential for *in vivo* applications.^52^ The development of SERS is therefore a significant expansion in the capabilities of Raman spectroscopy for bioanalytical applications and, in particular, for cancer diagnostics and in monitoring the treatment of cancer.^53–57^

An additional advantage of SERS is that, due to the sharp peaks present in Raman spectra, the technique is capable of detecting multiple targets simultaneously.^58–60^ An early example of multiplexing was demonstrated by Faulds *et al.* who were able to detect 6 DNA sequences corresponding to different strains of the *Escherichia coli* bacterium that were labelled with different commercially available dye labels.^61^ A SERS-based assay was also developed for the multiplexed detection and quantification of three bacterial meningitis pathogens with picomolar detection limits,^62^ and for genotyping human papilloma virus (HPV) from plasmid, cell line and clinical material with the ability to differentiate between six HPV genotypes.^63^ Furthermore, the simultaneous isolation and detection of three different bacterial pathogens has been achieved using SERS nanotags functionalised with antibodies specific to each target, demonstrating the capability of SERS for providing rapid and sensitive discrimination from a single sample.^64^ This shows the potential of the technique for advancements in biomedical applications and in future point of care devices, such as lateral flow immunoassays.^65^ Multiplexed detection using SERS has also been extended to cancer biomarkers.^66–69^ This includes the sensitive and simultaneous detection of multiple microRNAs associated with lung and breast cancer for the early diagnosis of the disease.^70–72^ This signifies the capabilities of the technique for cancer detection and diagnosis, where the simultaneous detection of multiple biomarkers is a significant advantage.

Despite the sensitivity of SERS and its multiplexing capabilities, along with the non-invasive and molecularly specific nature of Raman scattering, Raman is limited by its depth penetration capability and spectra of tissue are dominated by contributions from the subsurface layers, limiting the clinical application of Raman spectroscopy. However, since the transmission of light through tissue is dependent on the wavelength, for example light with a wavelength of 440 nm can only penetrate around 1 mm compared to 5 mm for near infrared (NIR) wavelengths (750 nm),^73^ the use of longer excitation wavelengths has improved the depth penetration of the technique. Confocal and purpose-designed instrumentation has also increased the applicability of the methods *in vivo*.^74,75^ Despite these improvements, it remains challenging to obtain spectral information from below the surface of the skin, without recourse to more invasive approaches such as needle probes.^76^ By applying an offset between the excitation and collection probes in a Raman experiment, photons scattered below the surface of the sample can be collected. This method, known as spatially offset Raman scattering (SORS),^77^ allows the collection of Raman spectra from depths significantly greater than those achievable using traditional confocal Raman microscopes, thus improving the potential of the technique for clinical applications.^78^ This has been validated by demonstrating the non-invasive analysis of bone^79–81^ and cancer tissue samples.^9,82^ By introducing nanoparticles into SORS experiments, the depth penetration capabilities of SORS can be combined with the sensitivity of SERS to allow Raman signals to be obtained from significantly increased depths through biological tissues. This alternative approach, known as surface enhanced spatially offset Raman scattering (SESORS), has allowed collection of Raman spectra from depths of around 5 cm through tissue samples.^83^ The advantages of SESORS for clinical applications have been demonstrated for glucose monitoring,^84^ detection of neurotransmitters through the skull,^85,86^ and for *in vivo* cancer imaging in live mice.^87^ The capabilities of SESORS for non-invasive detection *in vivo* is a significant step towards the application of Raman spectroscopy for the clinical diagnosis of cancer and in monitoring the effectiveness of treatment. This is a further demonstration of the versatility of Raman spectroscopy techniques and their potential in medical diagnostics.

One of the limiting factors in the clinical application of Raman spectroscopy, particularly SERS, is that a standard method is yet to be adopted and results can sometimes be considered irreproducible. Large inter-laboratory studies have recently been undertaken in an effort to overcome these issues,^88,89^ and recommendations have been published on the key parameters that should be considered to improve comparability of results across laboratories.^90^ These considerations are essential for clinical translation of the techniques and collaborative studies should continue such that standardised methods can be developed. Additionally, further use of SERS alongside clinical trials is required to prove the capabilities of the technique for cancer detection and diagnosis and so that the full potential of the technique can be realised.^91^

This feature article discusses the use of Raman spectroscopy for the detection, diagnosis and treatment monitoring of cancer and the progress of the technique towards clinical application, highlighting the research of our group in this area. The versatility of Raman spectroscopy allows the application of the technique in its various forms to the many approaches of studying cancer, from cellular imaging and biomarker detection to *in vivo* analysis. Here we discuss some of these approaches, demonstrating advances in Raman spectroscopy that provide benefits for the different methods and improve the potential of the technique for the detection, diagnosis and monitoring of cancer.

## 2. Raman imaging for cancer detection

To gain insight into the biochemistry of a cancer cell, cellular components can be identified using molecular biology-based approaches such as polymerase chain reaction, electrophoresis and Western blotting.^92,93^ They offer high levels of chemically specific information but require the cell to be lysed, which can introduce chemical modifications to the results. An attractive alternative is to Raman image cells to provide rapid, non-invasive and high spatial resolution of biochemical and structural information. However, as explored by Butler *et al.*,^94^ careful consideration of sample preparation, instrumentation, acquisition parameters and data processing must be taken into account in order to produce high quality data for analysis of biological material. Raman imaging has been used extensively to investigate biological changes. These include classification of different types of liver cancer and their proliferation states,^95^ investigating the uptake, distribution and metabolism of drugs in colon cancer cells,^96^ and to help understand the response in cancer cells when exposed to ionising radiation.^97^

### 2.1 Lipid imaging

**Fig. 1:**
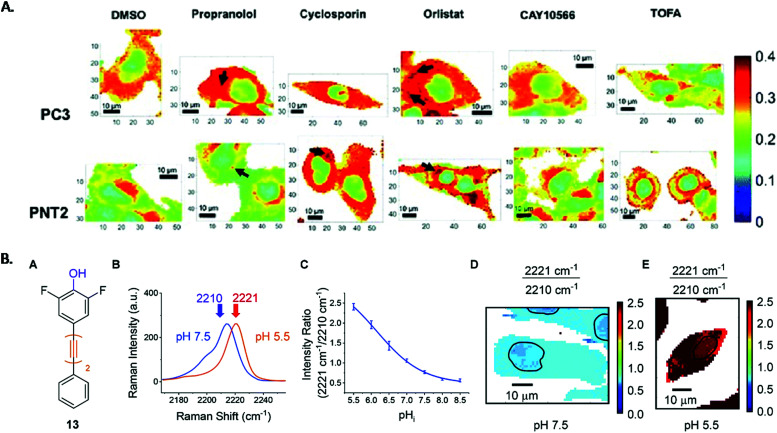
(A) Ratiometric Raman images of intracellular lipid distribution of PC3 and PNT2 cells treated with DMSO (control) and a lipid altering drug. False colour images are the ratio of the peak intensity at 2851 cm^−1^ and the sum of the peak intensities at 2933 cm^−1^ and 2851 cm^−1^, which reflected the lipid/(protein + lipid) ratio. Visual examination allows comparison between cancerous and non-cancerous cells to be made.^30^ B. Experimental procedure of intracellular pH sensing (A) structure of phenol, (B) spectra from PC3 cells at pH 7.5 (blue) and pH 5.5 (orange), (C) calibration curve of phenol in PC3 cells created using 2221/2210 cm^−1^ intensity ratio from PC3 cells, (D) and (E) false colour images of PC3 cells at pH 7.5 and pH 5.5 created using 2221/2210 cm^−1^ ratios. Nuclear regions highlighted by blank band.^102^ Reproduced from ref. 30 and 102 with permission from the Royal Society of Chemistry.

Lipids are an important cellular component whose intracellular uptake, distribution and metabolism are tightly regulated in healthy cells. However, these processes are disrupted in cancer due to the upregulation of *de novo* lipid syntheses.^98^ In order to develop new treatments, it is vital that the lipid biochemistry is understood. Raman analysis of prostate and bladder tissues indicated that the relationship between lipids and carcinogenesis could be measured.^99^ High resolution cellular Raman imaging built on this significantly by showing where these changes to cell biochemistry occurred, and it has been shown to give a detailed, high resolution insight into lipid distribution in cancer cells.^28,100^ Conventionally, the Raman peak intensities of the lipids is used to create Raman images, however recently ratiometric analysis of Raman peaks from cellular information in the fingerprint region has been shown to reflect lipid/protein abundance across a HEK293T cell.^101^ The ratiometric images were generated using the intensity ratio of 1448 cm^−1^ (which is associated with long aliphatic chains present in lipid species) divided by the sum of 1657 cm^−1^ (amide 1 vibrations) and 1448 cm^−1^. The images highlighted the nuclear region with lower lipid/protein content compared to the cytoplasmic region, reflective of the nuclear function to store DNA. This work was advanced by Jamieson *et al.* who used ratiometric values to build Raman images of intracellular lipid distribution of cancerous (PC3) and non-cancerous (PNT2) prostate cells, treated with drugs known to inhibit the enzymes involved in *de novo* lipid synthesis.^30^ To create a bivariate descriptor, the ratio between the high wavenumber region, 2851 cm^−1^ (C–H stretch in CH_2_ groups) and 2933 cm^−1^ (C–H stretch in CH_3_ groups), was selected to correlate lipid abundance. The false colour images of intracellular lipid distribution, shown in Fig. 1A, revealed a difference between cancerous and non-cancerous cells and a uniform distribution of lipids throughout the cytoplasm in PC3 cells compared to lower levels of lipid for PNT2 cells. The cells were then treated with three drugs that interfere with the different stages of *de novo* lipid synthesis and ratiometric Raman images of the lipid distribution created. Orlistat, an inhibitor of fatty acid synthase, elicited a phenotypic response characteristic of lipid accumulation in both cell lines. CAY10566, an inhibitor of the enzymes stearoyl-CoS desaturase (SCD) which creates mono-unsaturated fatty acids from saturated fatty acids, gave little response. Finally, 5-(tetradecyloxy)-2-furoic acid (TOFA), which inhibits the conversion of acetyl CoA to malonylCoA (one of the first steps in *de novo* lipid synthesis) induced a decrease in lipids, particularly in the cancer cells. The effect of two control drugs, cyclosporin and propranolol, which are capable of inducing the formation of lipid droplets were also investigated. Interestingly, propranolol showed selectivity towards cancerous cells, indicating it was a strong candidate to be investigated for selective anti-tumour action. It is clear that this non-destructive label free ratiometric analysis, performed using cost effective glass substrates, could revolutionise the understanding of drug–cell response and is a significant step in the monitoring of cancer treatment.

### 2.2 Alkyne imaging

An easily exploited region in the Raman spectra of cells is the ‘cell silent’ region (1800–2600 cm^−1^). Designing molecules that give a Raman band in this region can allow their distribution in cells to be easily tracked without any interference from the Raman signal of cellular components. Alkyne tags have become an important functional group as they give a strong band in the silent region (roughly 2120 cm^−1^).^104^ The first example of alkyne detection in cells was achieved using EdU (5-ethynyl-2′-deoxyuridine), a thymidine analogue with an alkyne group.^46^ EdU is incorporated into cellular DNA during DNA replication and accumulates in the nucleus. Due to the presence of the alkyne group, its location can be imaged using Raman mapping, demonstrating the potential of the alkyne moiety as a Raman tag in live-cell imaging of small molecules. The distribution of fatty acids tagged with an alkyne group has also been monitored and relative quantification was achieved, demonstrating how minimally invasive this technique is and how a small Raman tag can produce a large response.^101^ Recently, we have measured intracellular pH in prostate cancer cells (PC3) by designing low molecular weight oligoyne compounds that exhibit a pH sensitive alkyne stretching frequency.^102^ To quantitatively determine the pH, calibration within the environment of interest was performed. PC3 cells were treated with the compound and fixed to a discrete pH value. The cells were then Raman mapped and false colour images created using the ratio of the signals at 2221/2210 cm^−1^ (the bands corresponding to the change in alkyne shift at different pHs). The results from this approach are shown in Fig. 1B. The ratio varied as a function of pH in the cells and the compound was then used to monitor and quantify changes in pH in response to drug treatments. Cells were treated with etoposide, which induced apoptosis and should coincide with a decrease in pH. Over time, the change in ratio indicated that the pH decreased, demonstrating that the compound could effectively monitor and quantify changes in pH of live cells in response to drug treatment. To improve spatial resolution, the live PC3 cells were also imaged using SRS microscopy by selecting 2933 cm^−1^ (protein), 2951 cm^−1^ (lipid), 2221 cm^−1^ (alkyne) and 2321 cm^−1^ (off resonance) channels. The 2221 cm^−1^ channel confirmed that the alkyne was distributed in the cytoplasm, demonstrating the compatibility of the probe for intracellular pH sensing. This highlights another approach that could be used for the monitoring of cancer treatment and in enhancing understanding of the disease.

## 3. SERS for cancer detection and treatment monitoring

### 3.1 Biomarker detection using SERS-based assays

**Fig. 2:**
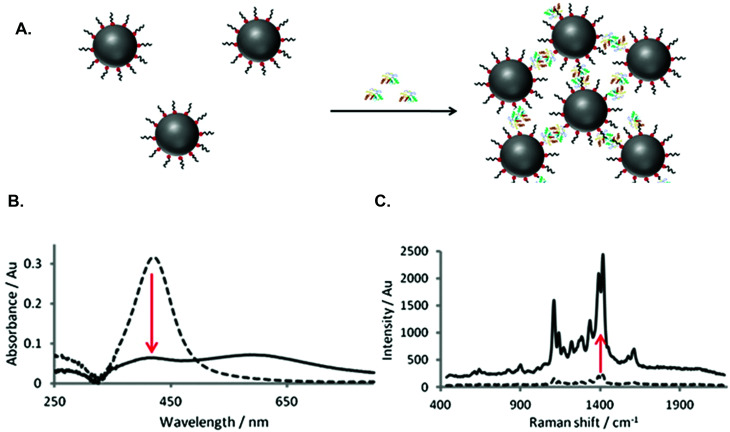
(A) Schematic of assay for MDM2 detection showing specific interactions between MDM2 and peptide on silver nanoparticle surface, resulting in nanoparticle assembly. (B) Change in extinction after nanoparticle assembly due to aggregation of nanoparticles. (C) Increase in SERS signal after nanoparticle assembly due to formation of hotspots.^115^

The detection of cancer biomarkers in body fluids overcomes the need for more invasive procedures, such as tissue biopsies. In addition, biomarker detection is more sensitive and specific than traditional morphological characterisation, thus potentially allowing detection of cancer at an earlier stage, increasing the PPV and therefore improving patient prognosis.^105^ For biomarker detection, SERS offers greater sensitivity than competing techniques and can also be used to detect multiple biomarkers simultaneously, allowing more accurate classification of cancer. With these advantages, it is unsurprising that SERS has been widely studied for the detection of cancer biomarkers and that various approaches have been explored.^106–108^ Early research in our group demonstrated that nanoparticles functionalised with biomolecules could be used to significantly enhance SERS signals by causing controlled aggregation of nanoparticles following specific biomolecular interactions.^109^ This nanoparticle assembly approach can be applied to various biomolecules and has thus been exploited extensively for the development of biological detection assays.^110–114^ Additionally, this method can be used to study biomolecular interactions, yielding significant information that may be useful in understanding cancer pathways. For example, the tumour suppressor protein, p53, plays a key role in many cancers and is regulated by mouse double minute (MDM2) protein. Therefore, understanding the interaction between these proteins could be invaluable in cancer therapeutics. Using a nanoparticle assembly approach with SERS detection, MDM2 interactions were studied in solution, allowing monitoring of the full protein, rather than focusing on only one binding interaction (Fig. 2).^115^ A p53-mimicking peptide was used to demonstrate the state of MDM2 in solution, while maintaining the biological activity of the protein. This approach validated the ability of SERS to study interactions of full length, unlabelled proteins using biologically driven nanoparticle assemblies, potentially aiding the understanding of biological pathways in diseases such as cancer.

SERS-based sandwich assays, which use capture antibodies bound to a surface and detection antibodies functionalised to a SERS nanotag, have also been explored in the group to detect low concentrations of clinically relevant biomolecules.^116^ This format has been exploited to detect cancer biomarkers including the detection of MUC4 expressed in pancreatic cancer,^117^ as well as the multiplexed detection of breast cancer^118^ and prostate cancer biomarkers.^117,119^ Cheng *et al.* used a SERS-based immunoassay for the simultaneous detection of two prostate specific antigen (PSA) markers and demonstrated the sensitive and specific detection of the biomarkers in clinical serum samples.^120^ They highlighted the potential applicability of the SERS-based assay for prostate cancer detection by comparing its performance to a current diagnostic assay.

### 3.2 Cancer cell imaging using SERS nanotags

Another popular approach of investigating cancer is to ‘tag’ biomarkers found on cancer cells followed by optical imaging. Conventionally, fluorescence tags are used as imaging agents; however, nanoparticles offer an attractive alternative due to their photostability, multiplexing capabilities, high spatial resolution, low background and enhanced sensitivity.^52,121^ By targeting cancer cells with SERS nanotags, cells can be analysed using Raman mapping experiments and the resulting SERS images can be used to differentiate between disease states, detect biomarkers on or within the cell, and assess the effectiveness of treatments.

Early cancer cellular nanoparticle incubation studies combined with SERS imaging did not target specific events, but focused on bare nanoparticle uptake *via* endocytosis. The cells were mapped and the resulting SERS spectra were indicative of changes in the chemical environment of the cell.^122^ In this label free approach, the spectra were complex and the analysis could be simplified by the inclusion of Raman active stains that allowed for faster mapping times. An example of this was demonstrated by Stokes *et al.*, who incubated bone-marrow-derived cells (macrophages) with gold and silver nanoparticles. The cells were then fixed, treated with a dye stain and analysed with line scanning SE(R)RS using biologically active wavelengths. Based on the SE(R)RS images produced by following a major peak of the dye throughout the cell, nanoparticle aggregates could be identified in secondary lysosomes.^123^ Raman signals of the dye were significantly enhanced due to their close proximity to the nanoparticle surface. However, it should be noted that the signal was only observed in locations where both the dye and the nanoparticle coincided within the cell and was not a true reflection of all the nanoparticles taken up by the cell. To increase the sensitivity of the approach SERS nanotags have been incubated with cells.^124–126^

A common predicament in nanoparticle incubation studies combined with SERS imaging is the question of whether the nanoparticles are actually inside the cell or merely bound to the cell surface. To address this, McAughtrie *et al.* demonstrated the first example of 3D SERS imaging for the simultaneous confirmation of the cellular inclusion and multiple component detection of SERS nanotags.^127^ Four SERS nanotags, labelled with different thiol-based Raman reporters, aggregated using 1,6-hexamethylenediamine (HMD) to create hotspots, were added to Chinese hamster ovary (CHO) cells. To verify nanotag uptake, the cells were 3D volume Raman mapped and 3D false colour SERS images were constructed by performing multivariate data analysis in the form of direct classical least squares (DCLS). Three out of four nanotags were located within the cells with spatial positioning. To employ SERS tags in cancer detection it is also important to assess their interaction and toxicity in cells. Bhamidipati *et al.* evaluated the toxicity of gold nanoparticles with different morphologies and surface chemistries and demonstrated that the surface chemistry had the predominant effects on cytoxicity, and that cetrimonium bromide (CTAB) coated gold nanoparticles were the most toxic and polyethylene glycol (PEG) coated gold nanoparticles the least.^128^

As well as inferring the location of SERS nanotags in cells, the Raman reporter can be used to investigate a variety of mechanisms that occur within the cell. For example, the activity of beta-galactose, a biomarker overexpressed in cancer, was detected in macrophages by monitoring the change in SERS signal that occurs when the reporter molecule 5-bromo-4-chloro-3-indlyl-beta-d-galactopyranoside, functionalised to gold nanoparticles, was hydrolysed by galactosidase to produce a SERS-active dimerised product.^129^ The change in SERS signal was visualised when the cells were Raman mapped and the resulting SERS image constructed using the large peak at 598 cm^−1^. The presence of the dimerised product inside the cells was evident, confirming the abundance of the enzyme. Cleavage of an alkyne Raman reporter, which can be followed ratiometrically, has also been utilised for the detection of caspase 3 in live cells with high sensitivity and good signal reproducibility.^130^ Caspase 3 plays a key role in apoptosis and has thus been used extensively as a cancer biomarker, particularly in monitoring prognosis.^131–133^

### 3.3 pH sensing and imaging

Homeostasis of intracellular pH is maintained at the organelle level under healthy conditions, but abnormalities can occur in cancer. To detect these changes faster and with increased sensitivities, nanotags and SERS measurements have been used. The pH sensitive Raman reporter 4-mercaptobenzoic acid (4-MBA) functionalised to a nanoparticle is conventionally used to build pH calibration curves based on changing peak ratios or intensities.^134^ The nanotags are then applied to a cell in numerous ways and the intracellular pH obtained. pH sensitive, SERS active fibre optic nanoprobes combined with Raman measurements were first used to measure the intracellular pH of human prostate cancer cells with no apoptosis nor aggressive lysomal response.^135^ In this example, the measurements were located to where the fibre optic was placed on the sample and did not give information on the cell as a whole or pH gradients within the cell. Subsequently Kneipp *et al.* attached 4-MBA to gold nanoaggregates and introduced them into mouse fibroblast cells before Raman mapping the cells.^136^ False colour plots of the calibrated ratio allowed the various pH values of the cell to be obtained. They displayed the dynamics of pH values in cells at sub-endosomal resolution. This approach has also been used for the SERS mapping of pH in live cells using 4-MBA functionalised to many different nanoparticles including silver clusters,^137^ gold nanoparticles,^138–140^ and gold nanostars.^141^ Building on the existing pH SERS mapping literature, Bando *et al.* paired pH SERS imaging with 3D nanoparticle tracking to trace the pH dynamics with a spatial accuracy of several tens of nanometres and a temporal resolution of 200 ms.^103^ By incorporating MBA onto self-assembled silver nanoparticles, nanogaps were designed for local pH sensing with high sensitivity, where the peak intensities of the carboxylate group (1390 cm^−1^) and CO stretching mode (1690 cm^−1^) showed a pH-dependent response. The assemblies were added to HeLa cells and time-lapsed SERS imaging showed time and location dependent pH changes in a living cell. This could be used to visualise the dynamic changes in the chemical environment caused by organelle interactions in cancer.

### 3.4 Biomarker detection and imaging in cells

Bioactive SERS nanotags have been successfully used as molecular imaging agents to target a number of biomolecules and chemical interactions specific to cancer. By incorporating a recognition motif onto the surface of a nanoparticle, the tag can target specific moieties on the surface of or inside the cancer cell and the interaction can be monitored by Raman mapping the cell and creating false colour images. For example, lectin-functionalised silver nanoparticles have been used to investigate carbohydrate–lectin interactions on the surface of mammalian cells.^142^ As there is an increase in sialic acid expression in malignant prostate cells, sialic acid-specific lectin conjugated SERS nanotags were used to discriminate between non-cancerous and cancerous cells. The nanotags were incubated with each cell type and Raman mapped, followed by the construction of false colour images by measuring the intensity of the main SERS peak from the benzotriazole dye. This allowed qualitative differentiation between the SERS signal from the cancerous cells, which produced a large SERS signal due to the lectin and sialic acid interaction and a very low signal on the non-cancerous cells. This successfully demonstrated that glycan expression can be correlated with malignancy using SERS. Various binding interactions have been investigated using this approach, including protein–ligand interactions accomplished using gold nanoparticles coated in RGDFC, a peptide that binds to the α_v_β_3_ integrin and is over expressed in colon cancer cells,^143,144^ folate receptor interactions on human ovary cancer cells, achieved by conjugating silver nanoparticles with folic acid,^145^ sentinel lymph nodes that were detected using ratiometric Raman dual-nanotag strategies using folate receptor targeted SERS tags,^146^ and for the detection of lymphoblastoid cells using silver coated gold nanoparticles conjugated to a DNA aptamer specific to the cell line.^147^

**Fig. 3:**
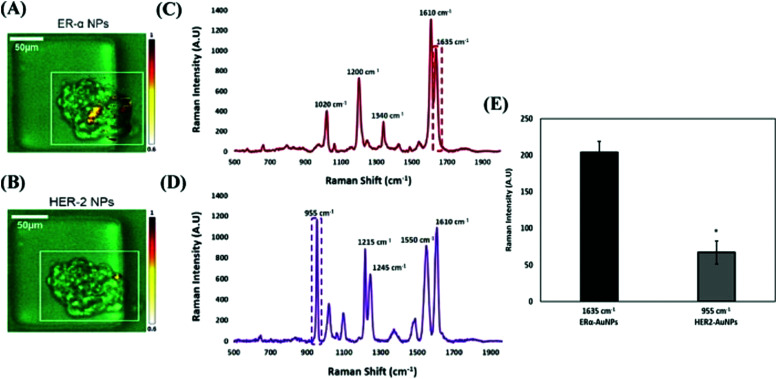
ERα-AuNPs showed a greater targeting effect and specificity for MCF-7 spheroids than HER2–AuNPs. MCF-7 spheroids incubated with the ERα + HER2–AuNP mixture (60 pM, 2 h) in microfluidic devices. The false colour images correspond to the SERS signal from (A) ERα-AuNPs and (B) HER2–AuNPs within the same spheroid. The minimum and maximum look up table (LUT) thresholds were set to exclude any poorly correlating or noisy spectra (minimum = 0.6). (C) Reference spectra of ERα-AuNPs (BPE Raman reporter) (red) and (D) HER2–AuNPs (PPY Raman reporter) (purple) in H_2_O. The spectra were collected using a 633 nm laser excitation, 100% laser power with 0.05 s accumulation time. The dashed box shows SERS intensity at 1635 cm^−1^ (red) that was selected as the representative peak for ERα-AuNPs (BPE Raman reporter) and SERS intensity at 955 cm^−1^ (purple) that was selected as the representative peak for HER2–AuNPs (PPY Raman reporter). (E) Average Raman intensities at 1635 cm^−1^ (ERα-AuNPs) and 955 cm^−1^ (HER2–AuNPs). The average of three samples from three independent biological replicates is shown. Error bars presented as mean ± S.D. * Significant difference (*p* < 0.05) in Student's *t* test.^149^

The most commonly employed recognition motif conjugated to nanoparticles are antibodies, which have been used to detect specific cancer related biomarkers. For example, the detection and identification of estrogen receptor alpha (ERα), which is one of the main biomarkers present in breast cancer, responsible for increased proliferation and metastasis, is crucial for the clinical diagnosis and correct treatment of the disease. Kapara *et al.* functionalised ERα specific antibodies to SERS nanotags that were then incubated with breast cancer cells and Raman mapped.^148^ The nanotags exhibited excellent biocompatibility along with spatial and temporal understanding of the location of the ERα location in breast cancer cell lines with different ERα expression status. To quantify the difference in cell lines, a sophisticated approach based on percentage of SERS response was used to determine that ERα positive breast cancer cells (MCF-7) exhibited a 4.2 times increase in SERS signal area in comparison to ERα negative cells (SKBR-3). This indicated the strong targeting effect of the antibody SERS nanotag towards the ERα. Furthermore, this method was used to investigate the activity of the drug fulvestrant, a selective estrogen receptor degrader (SERD). SERS mapping confirmed a weaker signal was obtained when cells were treated with fulvestrant due to ERα degradation, opening up the possibility of using SERS as a tool for the estimation of ERα expression levels. This work was expanded by employing the ERα specific antibody SERS nanotags for the detection of ERα expression in a 3D tumour model to better understand whether targeted nanotags are required to efficiently target ERα, or whether untargeted uptake by the EPR effect is sufficient.^149^ Using 2D and 3D SERS measurements, we successfully demonstrated the strong targeting effect of ERα specific antibody SERS nanotags, which had 63% more signal when compared to the non-targeted human epidermal growth factor receptor 2 (HER-2) specific antibody nanotags, confirming the differentiation between targeted and non-targeted nanotags (Fig. 3). Fulvestrant was also investigated in the 3D tumour model and ERα expression was again reduced, as confirmed by the lower SERS signal. This work highlighted the importance of performing assays on 3D cell cultures, which better reflect the tissue architecture and cell-to-cell/cell-to-matrix interactions present in real tumours. It also demonstrates the potential of using SERS nanotags to monitor ERα expression, with potential to be used for developing personalised treatment using primary cancer cells from patients.

One of the most promising advantages of SERS nanotags in cancer imaging is the multiplexing potential achieved by bio-conjugating SERS nanotags. Detection of multiple biomarkers is possible due to the narrow bandwidths of the reporter molecule, which can be imaged from the Raman maps to indicate the presence and location of multiple biomarkers within or on the surface of a cell. The rapid and sensitive phenotypic markers expressed on the cell surfaces of three different types of breast cancer cell lines have been detected using hollow gold nanospheres conjugated with specific antibodies.^67^ The results showed a quantitative distribution of the marker proteins as well as the cancer cell phenotypes *via* the SERS-mapping images. The simultaneous detection of two cancer biomarkers (MUC1 mucin and nucleolin) has also been achieved on the surface of MCF-7 cells using the self-assembly of branched DNA-gold nanoaggregates, providing information on the physiological and pathological states of the cancer cells.^150^*In vivo* cancer detection by SERS was first demonstrated by Maiti *et al.* who used antibody functionalised nanotags to target tumour sites in a mouse.^125^ The sites were Raman mapped and the resulting images revealed the location and distribution of each nanotag.

From these examples, it is clear that SERS has increased the sensitivity and selectivity over normal Raman for the detection of cancer in solution, surface and cell-based assays. This demonstrates the potential of SERS to be used as a pre-clinical screening technique that will detect cancer earlier and could fast track patients into treatment.

## 4. Raman and SERS analysis during clinical investigation and cancer surgery

Raman spectroscopy has been utilised for clinical investigations due to it being non-destructive, non-invasive, and having the ability to monitor changes in molecular composition in a biological sample, which could be indicative of disease. It has a number of other advantages including utilising a back scattering optical configuration, allowing measurements to be taken from below the surface in thick tissue sections without the need for micro-sectioning.^5^ Water is not a strong Raman scatterer and measurements can be taken in aqueous environments by using visible or NIR excitation to reduce the absorption effects of water. It also provides real-time molecular information at a relatively low cost. However, the technique lacks sensitivity due to the intrinsic weakness of Raman scattering, which can result in long acquisition times.^151^ Issues can also occur when using visible excitation sources, which decrease the depth of penetration, give rise to tissue autofluorescence and can cause issues due to heat generation.^152^ In addition, sophisticated data analysis is often required to deconvolute the complex signals acquired.^151^ New strategies are being developed to overcome some of these limitations, including using NIR excitation sources, carrying out spatially offset measurements and endoscopes combined with SERS measurements.

Endoscopic imaging is regularly used in clinical diagnostics as it is a minimally invasive method of examining tissues within the body. Endoscopic Raman spectroscopy, as opposed to white light imaging, can provide biomolecular information and enable objective diagnosis to be made.^153^ Pioneering work by Molckovsky *et al.* studied the diagnostic potential of NIR Raman spectroscopy of the colon and evaluated its ability to distinguish between adenomatous and hyperplasic polyps using a custom-built, fibre optic, NIR endoscopic system.^154^ Biochemical monitoring of the human cervix throughout preganacy,^155^ diagnosis of dysplasia in Barrett's esophagus,^156^ gastric cancer diagnosis,^157^ and early lung cancer detection^158,159^ have also been investigated using endoscopic Raman probes.

To increase the sensitivity of the approach, it has been combined with nanoparticles and SERS measurements. A Raman endoscopic probe was designed by Zavaleta *et al.*,^160^ who inserted the device through a clinical endoscope and demonstrated the multiplexed detection of tumour-targeting nanoparticles. Jeong *et al.* developed an endoscopic device that combines fluorescence and Raman and used the technique for the simultaneous *in vivo* detection of cancer biomarkers, HER2 and EGFR, in breast cancer tissue.^161^ A novel, non-contact, opto-electro-mechanical device was also developed for the rapid imaging of large areas in the human gastrointestinal tract.^162^ This approach was also capable of detecting multiple SERS nanoparticles simultaneously, and showed potential for cancer diagnosis and treatment monitoring. Evidently, the combination of Raman spectroscopy with endoscopy is a useful approach for investigating cancer and potentially monitoring treatment. Alternatively, Raman spectroscopy can be used during surgery to guide procedures and aid successful resection. Karabeber *et al.* showed that by injecting tumour-bearing mice with silica-coated gold nanotags, accumulation of the nanoparticles occurred in the brain tumours and could be detected using SERS.^163^ This allowed imaging of the tumours using a handheld spectrometer that aided the removal of the tumour and showed improved resection when compared to surgical guidance using white light imaging. In a further development, Jermyn *et al.* developed a handheld Raman probe and demonstrated its use during live human brain surgery.^16^ Using an NIR laser and placing the fibre probe in contact with the brain tissue, they could differentiate between normal and cancerous cells in the human brain with greater accuracy (92%) than alternative techniques such as microscopy and MRI (73%). Wang *et al.* applied SERS-active targeting nanotags to freshly excised human breast tissue and obtained quantitative multiplexed molecular imaging in only 15 minutes, indicating that this approach could be used for guidance during breast cancer surgery.^164^

These are just some of the examples where the advantages of Raman spectroscopy have been exploited for the detection and diagnosis of cancer in a clinical environment. Evidently, further work is required before the techniques will be adopted in medical clinics; however, the potential of the methods has been demonstrated across several areas of cancer detection, using several different approaches.

## 5. Gaining depth in cancer detection *via* SORS and SESORS

The advantages of SERS for sensitive, specific and multiplexed detection can be further driven towards clinical applications by allowing the non-invasive detection of lesions buried beneath the surface of the skin. Spatially offset Raman scattering (SORS) and surface-enhanced spatially offset Raman scattering (SESORS) are novel methods that enable *in vivo* detection of the molecular changes associated with diseases, such as cancer, by facilitating the ability to obtain signals from depths up to several centimetres below a surface. This allows non-invasive monitoring of signals from tissues *in vivo*, which could significantly improve early cancer detection and treatment monitoring.

By offsetting the signal collection probe from the laser excitation probe in Raman spectroscopy, photons scattered from the subsurface medium can be collected, allowing signals to be obtained from below the surface and through barriers, such as tissues, with an increasing offset resulting in signals being obtained from greater depths.^165,166^ Since the first demonstration of SORS in 2005,^77^ the technique has been successfully applied for the transcutaneous *in vivo* analysis of human bone^79^ and the through tissue analysis of tumours^167^ and calcifications^82^ in breast tissue, indicating its potential for non-invasively detecting cancer in its early stages.^82^ The capabilities of SORS for clinical applications have also been highlighted by demonstrating that signals can be obtained from significant depths, through-barrier, using a handheld spectrometer.^166^ Although this study focussed on the detection of ethanol through plastic, it showed the potential of using both conventional Raman and SORS in clinics, where handheld spectrometers would be particularly advantageous, and verified that signals can be obtained from greater depths when using SORS than by focussing into the sample using normal Raman optics.

The potential of SORS is further enhanced by combining its capabilities with the sensitivity of SERS to achieve significantly improved signals from even greater depths, as well as introducing the ability to target specific disease markers using tagged nanoparticles. SESORS was first proposed in 2010,^83^ when it was established that SERS nanotags could be detected through 25 mm of porcine tissue using transmission Raman, where the collection probe was placed on the opposite side of the sample to the laser. Transmission Raman is an example of an extreme spatial offset, where the angle between excitation and collection is 180°. Silver nanoparticles functionalised with a NIR dye were injected into tissue samples and the potential of the technique for the detection of small tumours was described, indicating the number of nanoparticles that may be required for the detection of lesions of particular sizes. In a further development, SESORS imaging was implemented and four different flavours of SERS nanotag were injected into a porcine tissue block, where their unique signals were non-invasely detected from a depth of 20 mm.^168^ False colour images were generated using the most intense peak for each flavour of nanotag and the spatial distribution of each nanotag could be observed. Signals were obtained from the nanotags at 47 mm; however, the signal deteriorated at the greater depth, particularly above 1250 cm^−1^, due to the increased absorption from water and myoglobin from the tissue in this region. In this study, nanotags were encapsulated such that the SERS signal was obtained from Raman reporters rather than target molecules; however, functionalisation of the nanotags with molecules of interest such as cancer biomarkers, cell specific proteins or DNA fragments would allow application of the technology for cancer detection and treatment monitoring. Bisphosphonate-tagged AuNPs were used to target calcium on the surface of bone samples, where the bisphosphonate/calcium binding enabled detection of the nanotags from the surface of the bone using Raman mapping.^169^ To demonstrate potential for *in vivo* imaging, bone samples covered in bisphosphonate-functionalised nanotags were covered with 20 mm of porcine tissue to mimic detection of the nanotag-functionalised bone through tissue. Spatially offset Raman maps were collected across the bone samples and principal component analysis (PCA) was used to identify the peaks from the nanotags and the bone. This demonstrated the detection of a fine distribution of NPs from the surface of bone, rather than a concentrated droplet injected into tissue. The use of bone/calcium specific nanotags to obtain a SESORS signal from the surface of the bone, through 20 mm of tissue, showed potential for detection of metastatic breast cancer, as well as bone disease.

**Fig. 4:**
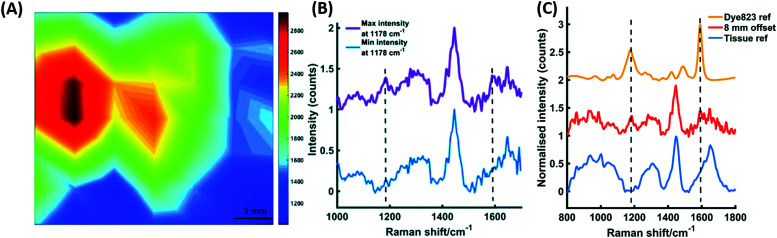
(A) A false colour *xy*-2D SESORRS heat map of MTS containing nanotags through 15 mm of tissue. The map was constructed using the peak intensity at 1178 cm^−1^. Measurements were carried out using an *xy* translational stage in step sizes of 3 mm to create an image of 7 × 7 pixels. (B) The corresponding maximum and minimum collected 8 mm offset spectra. (C) The tracking of nanotags through 25 mm of tissue. The tissue and dye reference spectra are shown at the bottom and top, respectively. The middle spectrum represents the Raman signal collected at an 8 mm offset through 25 mm of tissue. The peak at 1178 cm^−1^ was easily detectable by eye and the peak at 1592 cm^−1^ was also detectable, albeit to a lesser extent. All measurements were carried out using a 2 s integration time, 5 accumulations, 830 nm laser excitation wavelength.^173^ Reproduced from ref. 173 with permission from the Royal Society of Chemistry.

One of the greatest advantages of SESORS is its potential for the non-invasive detection and monitoring of tumours *in vivo*. Multicellular tumour spheroids (MTS) are used as tumour models to mimic the 3D *in vivo* environment of tumours. This allows the *ex vivo* study of cancer, closely mimicking the *in vivo* environment, without the need for ethical approval and more complicated experiments. Nanoparticles are known to passively accumulate in tumours, allowing SERS imaging to distinguish between cancerous and healthy cells.^170,171^ MTS can be grown with uniformly distributed NPs to mimic the accumulation in tumours and thus provide a model for *ex vivo* tumour detection.^172^ This has been utilised to demonstrate the use of surface enhanced spatially offset resonance Raman scattering (SESORRS) for imaging a live breast cancer tumour model through tissue using a handheld spectrometer.^173^ SESORRS involves the incorporation of a dye-label with an electronic transition close to the frequency of the exciting laser, to significantly improve the sensitivity and therefore depth penetration of SESORS.^174^ Human breast cancer cells were incubated with resonant dye-labelled AuNPs resulting in the accumulation of the nanotags within the cells, which were then used to grow MTS.^173^ The MTS were then transferred to a section of tissue and a 15 mm section of porcine tissue was placed on top of the layer to simulate the detection of SERS nanotags through the tissue using SORS. Spectra were acquired from the MTS models by probing the tissue sample using a handheld SORS instrument, with an 830 nm laser excitation wavelength in backscattering configuration and an 8 mm spatial offset. Peaks in the spectra at 1178 cm^−1^ and 1592 cm^−1^ corresponded to the dye label, demonstrating the uptake of the nanotags into the MTS. Spectra were collected every 3 mm to create an image with 7 × 7 pixels and a false colour map was generated based on the intensity of the peak at 1178 cm^−1^ (Fig. 4(A)). The location of the MTS models is evident in the areas of maximum intensity and the signal from the MTS is clearly distinguishable from the background tissue signal (Fig. 4(B)). This gives an indication that SESORRS imaging could be used to detect functionalised nanoparticles through 15 mm of tissue, thus demonstrating the potential of the technique for *in vivo* tumour detection. The capability of this approach was further validated by analysing SERS nanotags through 25 mm of porcine tissue (Fig. 4(C)). Again, using an 8 mm offset with the handheld SORS instrument, signal could be obtained from the SERS nanotags through the tissue, and peaks from the dye at 1178 cm^−1^ and 1592 cm^−1^ were clearly distinguishable from the tissue reference. Although greater depth penetration was achieved previously,^168^ this was using a transmission geometry on a benchtop SORS instrument. In the work described here, backscattering geometry was used, where collection is from the same side of the sample as the exciting laser but with a spatial offset applied, rather than collecting from the opposite side of the sample. The use of a handheld spectrometer with backscattering optics signifies the potential of this technique for clinical applications and was a significant step towards the non-invasive detection of tumours.

In a further development, a similar approach was used to demonstrate the multiplexing capabilities of SESORRS.^175^ The detection and classification of three nanotags, both individually and as a triplex, was performed through 10 mm of tissue using handheld SESORRS. Spectra were collected from the three individual dyes and from a mixture of the three at equal concentrations, both from a MTS tumour model and from nanotags in solution. Since the Raman spectra of the three dyes were fairly similar, PCA was applied to discriminate between the single nanotags and the triplex. The resulting scores plots gave clear separation into four distinct groups for the three individual dye spectra and the spectra of the triplex, demonstrating the successful identification and discrimination of single and multiplexed SERRS nanotags through 10 mm of tissue using a handheld SORS spectrometer. This highlights the potential to simultaneously detect multiple targets *in vivo*, which is advantageous for the detection and monitoring of disease, where the sensitive detection of multiple biomarkers is of significant interest to determine cancer phenotype.

The recent developments in the through tissue detection of live breast cancer tumour models exploited the enhancement in signal that can be achieved by using a dye that is in resonance with the laser excitation wavelength.^173,175^ This resonance effect allows significant enhancement in SERS signal, which in turn enables greater depth penetration. This concept was further examined by comparing the signals obtained from nanotags functionalised with a non-resonant reporter (SERS tags) to those observed when functionalised with a resonant dye (SERRS tags).^174^ Observed detection limits were 11 times lower when the resonance effect was exploited and a calculated detection limit of 104 fM was suggested when using SESORRS. Detection of nanotags using handheld instrumentation at this level of sensitivity, through clinically relevant depths, shows the potential of SESORRS for clinical applications and for *in vivo* detection of cancer.

An early demonstration of the potential of SESORS for *in vivo* detection was the transcutaneous detection and quantification of glucose *via* implanted silver film over nanosphere (AgFON) surfaces.^84,176^ Using a capture layer of decanethiol/6-mercapto-1-hexanol (DT/MH), glucose was attracted to the AgFON surface, where its Raman signal was enhanced, and could be detected through skin using SESORS. The sensor proved to be functional for 17 days after implantation, with high accuracy and consistency, using laser powers that are safe for skin exposure. The capabilities of SESORS for *in vivo* detection were further demonstrated by Sharma *et al.*, who obtained spectra of nanotags embedded in tissue through bone.^177^ This was the first demonstration of through bone detection using SESORS, which demonstrated the potential of the technique to be used for through-skull analysis. This was later proven when SESORS was used for the non-invasive detection of neurotransmitters in a brain tissue mimic through a cat skull.^85^ The detection of melatonin, serotonin and epinephrine was achieved down to concentrations of 100 μM and PCA was used to demonstrate that unique spectra were obtained from each of the three neurotransmitters.

Nicolson *et al.* recently reported the first use of *in vivo* SESORRS imaging to obtain Raman spectra from brain tumours in mice through the skull.^87^ Au nanostars were tagged with a resonant Raman reporter to create SERRS nanotags that were then functionalised with a cyclic RGDyK peptide, to enable specific targeting to glioblastoma multiforme (GBM) tumours *in vivo*. The RGD-SERRS nanotags were injected into the tails of five tumour bearing mice and prior to imaging the mice were anesthetized. SESORRS images were collected from the mice and compared with conventional Raman images. Using the SORS setup, stronger Raman signals were obtained and a greater tumour to background contrast was observed. This allowed tumour location information to be resolved using a lower laser power, indicating the applicability of the technique for *in vivo* clinical applications.

## 6. Raman in the clinic

The work presented in this review indicates how Raman spectroscopy has the potential to become an important clinical tool in cancer detection, diagnosis and treatment monitoring. However, despite great promise, its translation into the clinic for widespread human use is slow. This is due to a number of challenges including safety, cost, sustainability, duration of analysis, laser source and power, and auto-fluorescent tissues.^152^ Perhaps the largest obstacle is simply demonstrating the added value that Raman analysis can provide over, or in combination with, existing technologies. Although a challenge, many research groups have investigated the potential of Raman spectroscopy for clinical use, mostly in the form of *in vivo* studies with patient samples, focusing on the sensitivity and specificity of the approach.^178,179^*Ex vivo* and *in situ* measurement on patients have also been achieved to differentiate between cancerous and non-cancerous specimens.^180,181^

SERS clinical translation also raises new challenges such as the synthesis of reproducible SERS tags and lack of clinical evaluation when measuring *in vitro* samples such as serum or blood.^182^ These issues are being addressed by synthesising SERS tags on a larger scale and introducing protecting agents such as mercaptoundecanoic acid to provide stability.^183^ More emphasis is also being placed on clinical evaluation involving testing of cohorts of well-characterised patient samples.^184–186^ Of course, there are also major barriers to administering nanoparticles *in vivo*, which need to be overcome before they can be safely and routinely used in humans. These include the toxicological effects,^187^ non-specific binding and formation of protein coronas on the nanoparticle surface,^188^ circulation time,^189^ clearance pathways,^190^ and labelled nanoparticles altering their physicochemical properties.^191^ In order to progress, biocompatible and biodegradable SERS probes with minimal cytotoxicity are being investigated and several groups have shown minimal toxicity with gold nanoparticles coated in a number of different protective layers such as silica^192^ and PEG.^193,194^

In a recently published review, Xi and Liang retrieved the number of Raman spectroscopy clinical trials being carried out from the International Clinical Trial Registry Platform (ICTRP) search portal using the key word ‘Raman’. The registered trials were then screened to exclude non-related Raman records or repeat studies.^195^ As of 2021, the search produced 55 registered Raman spectroscopy clinical trials, with 36% currently recruiting. The trials can be split into 5 categories: 54.5% are ‘observational’ aiming to observe patients to measure certain outcomes without intervention; 32.7% are ‘interventional’ which evaluate one or more particular intentions; 9.1% are ‘diagnostic’, evaluating diagnostic accuracy; 1.8% are meta-analysis, a statistic process combining findings from individual status; and 1.8% are ‘relevant factors research’. There are also 6 SERS clinical trials, one of which is aimed at detecting circulating tumour cells in peripheral blood originating from breast cancer tissue.^196^ It should be noted that in 83.6% of the trials, the recruitment sample size is less than 200 subjects and it has been suggested that university and research centres need to forge a more collaborative effort with clinicians and industrial sponsors to carry out large-scale, high quality and multicentre registered Raman clinical trials.

A search of published clinical trials was also performed in PubMed by searching for ‘Raman’ with the article type restricted to ‘clinical trials’. This search resulted in 44 published clinical trials using various Raman techniques, with confocal Raman and transcutaneous Raman being approved to meet the clinical accuracy requirement for the non-invasive detection of glucose *in vivo.*^197,198^ However, the majority of these studies had sample sizes of less than 100 and were carried out at single sites. This lack of consistency could deter investors, hamper product development and delay translation.^199^ To address this, multicentre studies and inter-laboratory ‘round robins’ need to be implemented to reduce bias, validate the robustness of the technique and generate more convincing evidence. It is evident that there are still barriers that Raman spectroscopy clinical trials need to overcome, however there are strategies that can be employed to produce clear, concise and compelling evidence that Raman should be used in the clinic. Although it is still not the ‘gold standard’, it is evident that Raman and SERS can offer tremendous gains in cancer detection, diagnosis and treatment, and that the outlook remains positive.

## 7. Conclusions and outlook

Raman scattering and its enhanced forms offer many advantages for use in cancer detection, diagnosis and treatment monitoring. Each of the different techniques have individual advantages, enabling applicability in the many different approaches of investigating cancer. While Raman spectroscopy can be used to give molecularly specific information that can be useful in determining disease states, variations in the technique can further improve its capabilities. For example, SRS can vastly increase imaging speeds at the cost of spectral molecular information, and the use of SERS significantly improves sensitivity while introducing the capability for targeted assays. Recent modifications, like SORS and SESORS, open up further opportunities for *in vivo* analysis by allowing spectra to be collected through tissue. Additionally, handheld SORS instruments and probe-based SERS systems have been developed and demonstrated for their potential use *in vivo*, making the techniques suitable for point of care testing. Despite the advantages and progression towards clinical application, the full potential of Raman spectroscopy is yet to be exploited for medical diagnostics. This is due to several factors mainly pertaining to the use of nanoparticles in the body that can have a toxic effect such as inducing oxidative stress or cellular damage, poor retention times that reduce their targeting properties, and unclear excretion pathways. Other issues, such as cost, analysis time, and difficulty proving the advantages over current standard methods, also reduce the use of unlabelled Raman spectroscopy in the clinic. For these reasons the clinical use of Raman spectroscopy is still limited; however, with recent developments allowing faster imaging speeds, improved sensitivity and greater *in vivo* potential, instruments with clinically safe laser powers can be used to non-invasively obtain quantitative and detailed information for the detection and diagnosis of cancer. In comparison to current optical imaging techniques, such as MRI or ultrasound, Raman spectroscopy can obtain more detailed biochemical information and is a more quantitative method of analysis. However, these techniques have been employed for many years and are widely accepted as being suitable for cancer detection and medical diagnosis in general. Concerns with, for example, the safety of using lasers in the clinic and toxicity of nanoparticles for human consumption, must be overcome before use in clinical practice will be considered. Therefore, larger studies are required to demonstrate that the instrumentation and methods are safe for clinical use. The various techniques discussed in this review allow the advantages of Raman spectroscopy to be exploited for the detection and diagnosis of cancer in many ways, from *in vitro* biomarker detection and *ex vivo* tissue analysis to *in vivo* tumour detection. This indicates that the different techniques and applications complement each other well and could provide a toolbox for medical applications. Further clinical studies are required to prove the benefits of the techniques but the area is moving in the right direction to achieve this and to move towards clinical translation.

## Conflicts of interest

There are no conflicts of interest to declare.

## Supplementary Material
